# Orthodontic treatment in times of Covid-19

**DOI:** 10.25122/jml-2021-0038

**Published:** 2021

**Authors:** Hariclea Morosan

**Affiliations:** 1. Private Dental Practice, Bucharest, Romania

**Keywords:** Covid-19 pandemic, orthodontic patient, delayed treatment

## Abstract

During the first two months of the Coronavirus Disease 2019 (Covid-19) pandemic, Romania was in lockdown, and all dental practices were closed, so orthodontic patients had to postpone their check-ups for at least eight weeks. This led not only to a delayed end of treatment but also to accidents and complications. The present study tried to evaluate the orthodontic situation both from the patient’s and orthodontist’s point of view, so the patients were given a few questions to answer, and the orthodontist analyzed each treatment before and after the two-month lockdown and decided if it was mildly or severely affected by the absence of check-ups. The study group consisted of 105 patients evaluated by three orthodontists in the same private practice. Patients that have gotten worse after the lockdown or who had problems were included in the study. Also, all the patients were given a 7-question form in order to find out their opinion. After two months without check-ups, our orthodontists found that 9.52% got worse because of the lack of intermaxillary elastics, broken brackets, broken removable orthodontic appliances, and others. Most of the patients believe that their treatment was delayed by the Covid-19 pandemic, but none of the patients felt unsafe when visiting the clinic. The Covid-19 pandemic had severe effects on orthodontic treatments. Orthodontists noticed a delay for about one-third of their patients. However, from the patient’s point of view, half believe that their treatment was negatively affected by the Covid-19 pandemic in different degrees.

## Introduction

The novel coronavirus disease triggered a worldwide epidemic which affected all population despite the protective measures that were taken [1–3]: lockdown, social distancing, use of personal protection equipment, disinfectants [4–6] and others.

In Romania, orthodontic patients were postponed for two months during the lockdown, meaning that they were not allowed to go to their monthly visit to their orthodontist because the dental practices were closed due to the Covid-19 pandemic. Some of these patients got even more delayed as they had the Severe Acute Respiratory Syndrome Coronavirus-2 (SARS-CoV-2) infection or have been a direct contact to a positive case of COVID-19 and needed to be quarantined for at least two more weeks. This situation was met in several countries in Europe as well [7–10].

There are many implications of this novel disease on orthodontics, but this research could not cover all the aspects, only the most obvious ones and the first ones to appear.

The present study aims to assess how the Covid-19 pandemic affected the orthodontic treatment of patients and evaluate their opinion on the matter, report how much the orthodontic treatment was delayed, damaged, or even gotten worse, especially in patients with fixed appliances.

## Material and Methods

The conducted study took place inside an orthodontic private practice in Bucharest, Romania. For this study, three orthodontists evaluated their given patients; the criteria were if and which patients have gotten worse after the lockdown, which stayed the same, and which had problems and complications like broken brackets or lack of intermaxillary elastics. All the data needed was collected into a table. The study group consisted of 105 patients, both children and adults.

The orthodontic patients or their parents were given a form consisting of 7 questions. No personal data was given, and the patients signed a consent to participate in the present survey. The questions are:

1. How did the Covid-19 pandemic affect your orthodontic treatment? Did you feel safe coming every month for a check-up during this pandemic?
2. Do you consider that the protection measures against Covid-19 from the orthodontic practice are necessary?
3. Do you think the doctor-patient relationship was negatively affected by wearing the personal protection equipment?
4. When do you think we will come back to normal (no masks and no social distancing)?
5. For parents: did you notice new vicious habits like nail-biting, lip/finger sucking of your child since the beginning of the Covid-19 pandemic?
6. Did you notice a predisposition towards teeth clenching and grinding during the daytime or at night since the beginning of the Covid-19 pandemic?
7. For the statistical processing of the data from the current study, Microsoft Excel (Microsoft Office 2015), Google Docs and Google Drive were used.

## Results

**Figure 1.**
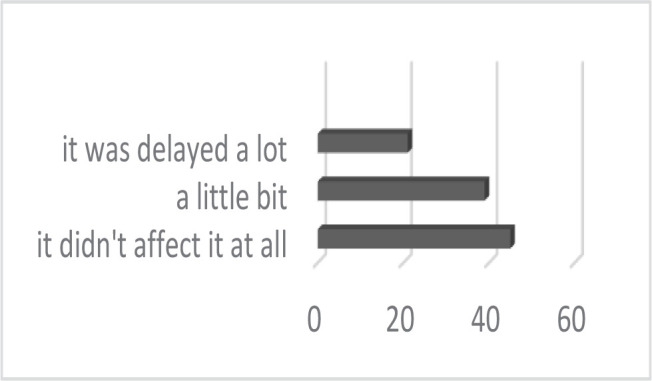
How did the Covid-19 pandemic affect your orthodontic treatment?

The study group included 105 orthodontic patients, 51 children, and 54 adults with orthodontic treatment started before February 2020. Three orthodontists from a private practice analyzed their patients before and after the two-month lockdown, and the data was centralized in Table 1. Regarding the patient’s opinion, 57.14% believe that their treatment was negatively affected by the Covid-19 pandemic in different degrees, as shown in Figure 1.

**Table 1.** Patients’ orthodontic situation after lockdown.

Patient’s age	Condition got worse	Condition stayed the same	Condition got better
**9–11**	2	7	18
**12–16**	1	4	13
17–20	3	6	17
21+	4	8	22
Total %	9.52%	23.81%	66.67%

**Figure 2.**
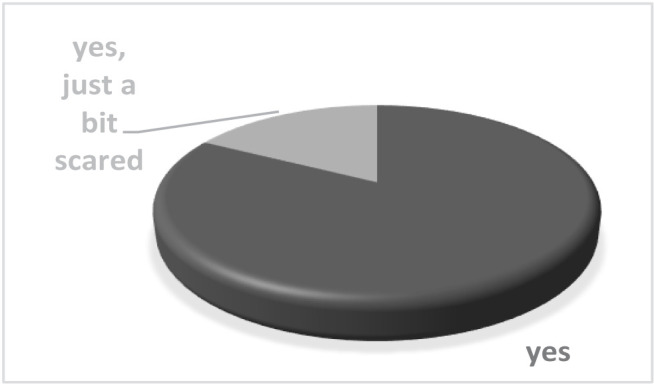
Did you feel safe coming every month for a check-up during the pandemic?

Most patients felt safe coming monthly to the orthodontic practice for check-ups (82.85%). Also, 17.15% were just a little bit scared, but none of them felt unsafe. The diagram is shown in Figure 2.

The supplementary measures taken by the private practice were considered necessary by 85.71% of the patients, while 5.71% believed they were not mandatory. The complete details are shown in Figure 3.

**Figure 3.**
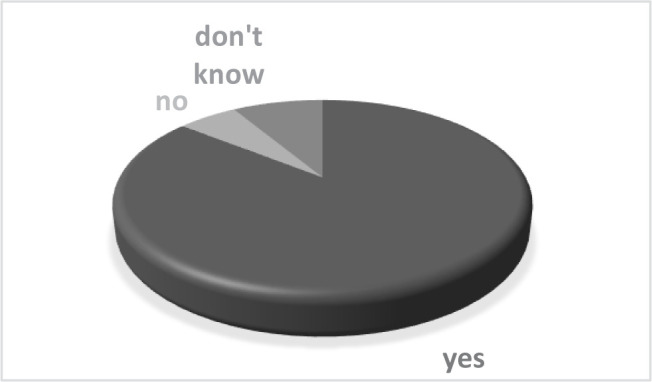
Do you consider that the protection measures from the orthodontic practice against Covid-19 are necessary?

The parents of children with orthodontic treatment believe that the doctor-patient relationship was not affected by wearing personal protection equipment in any way. We need to take into consideration the fact that all these children established a relationship with their doctor before the Covid-19 outbreak when they could see each other’s faces, and the doctor did not wear all the protection equipment.

**Figure 4.**
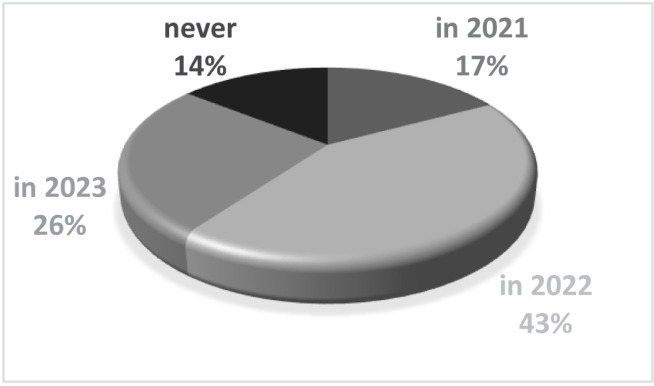
When do you think we will come back to normal (no masks and no social distancing)?

Trying to look for positive thinking among patients, we discovered that most of them (42.85%) believe that we will be returning to normal by 2022. All the different opinions are listed in Figure 4.

**Figure 5.**
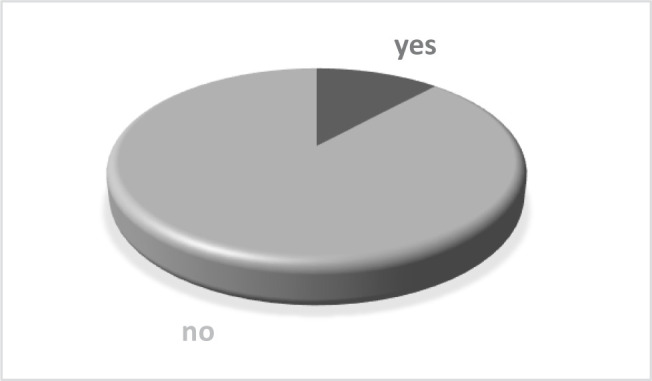
Did you notice new vicious habits (like nail-biting, lip/finger sucking) of your child since the beginning of the Covid-19 pandemic?

Our present study also included 51 children, for which we tried to assess the level of stress expressed through vicious habits that may have been noticed from the beginning of the Covid-19 pandemic. When being asked, only 11.76% of parents reported new oral habits in their children (Figure 5).

We also tried to measure the stress level to which the adult orthodontic patients were exposed during this global situation, and we noted the clenching and teeth grinding that occurred since the beginning of the Covid-19 pandemic. 17.14% of the adult orthodontic patients started grinding and clenching teeth after the Covid-19 outburst (Figure 6).

**Figure 3.**
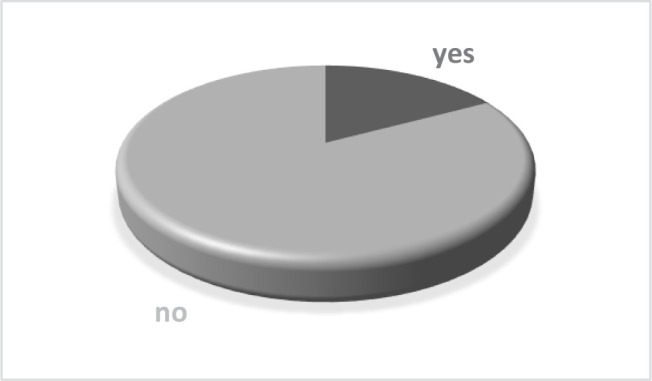
Did you notice a predisposition towards teeth clenching and grinding during daytime or at night since the beginning of the Covid-19 pandemic?

## Discussion

On a regular basis, each month, the orthodontists notice an improvement in occlusion, teeth alignment for all their patients as a result of the orthodontic treatment they are conducting. However, last spring, patients were postponed for two months because of the lockdown, being in touch with their orthodontist only by telephone or internet [11–13]. This situation had a few implications which, in our clinic, were centralized as follows: only 66.67% of the orthodontic patients got better after the 2 months without check-ups, while 9.52% got worse because of the lack of intermaxillary elastics, broken brackets, broken removable orthodontic appliances and others. This situation poses a serious question mark to the orthodontic management of patients through a period of crisis like this one [14–16].

Most of the patients believe that their treatment was delayed by the Covid-19 pandemic – 57.14% – a rather small number, considering that all patients included in this study had started their orthodontic treatment before February 2020. From the specialist’s point of view, it is evident that for all of them, two months without check-ups means for sure at least a delay in results. On the other hand, many patients might have been at the beginning of their orthodontic treatment when the alignment of teeth was still incomplete, so during the lockdown, the teeth “took their time” to align fully.

During these last pandemic months, the orthodontists tried to make the dental practice a safe place for the patients as the new coronavirus is known to be spread through aerosol particles [17, 18], so it is a joyful moment to find out that none of the patients felt unsafe when visiting the clinic for monthly check-ups. However, a small part of the patients, 17.15%, felt safe but also a little bit scared – a normal reaction if we take into consideration all the official data, which was quite imprecise at some point [3, 20].

Most of the questioned patients considered the new regulations for protection against Covid-19 as necessary, meaning they understood why they sometimes have to wait outside the clinic not to overcrowd the waiting room or use hand disinfectant each time they enter the office [21].

Most of the patients in our private practice (43%) believed that things would be coming back to normal in 2022, so they keep their positivity level up, considering that specialists hope that it will happen by 2023 [5–7]. However, there is a small percentage of 14% who think that we will never come back to normal, showing not only the lack of hope but also the fact that they have accepted this situation.

It is well known that during this last pandemic year, the stress level for everybody went high [8–10, 19]. Our measuring tool was the vicious oral habits. Surprisingly, there was no significant difference between children and adults, and their new oral habit rates were lower than we expected - 17% for adults and 11.7% for children. This fact might show that most of the questioned patients found other ways to cope with the stress during this period.

## Conclusion

The Covid-19 pandemic had severe effects on orthodontic treatments; orthodontists noticed a delay for about one-third of their patients. However, from the patient’s point of view, half believe that their treatment was negatively affected by the Covid-19 pandemic in different degrees. Almost all orthodontic patients found a safe place when coming to the orthodontist’s office every month, meaning they trust their doctor and believe that the new regulations are effective.

## Acknowledgments

### Consent to participate

Written informed consent was obtained from the participants.

### Conflict of interest

The authors declare that there is no conflict of interest.
